# ﻿A new species of genus *Crenotia* (Bacillariophyta) from Tibet, China

**DOI:** 10.3897/phytokeys.237.112939

**Published:** 2024-01-11

**Authors:** Xinyuan Na, Jiaming Liu, Ying Zhang, John Patrick Kociolek, Maxim Kulikovskiy, Xinxin Lu, Fengyang Sui, Huan Zhu, Guoxiang Liu, Yawen Fan, Yan Liu

**Affiliations:** 1 College of Life Science and Technology, Harbin Normal University, Harbin, 150025, China Harbin Normal University Harbin China; 2 Museum of Natural History and Department of Ecology and Evolutionary Biology, University of Colorado, Boulder, CO 80309, USA University of Colorado Boulder United States of America; 3 K. A. Timiryazev Institute of Plant Physiology RAS, IPP RAS, Moscow 127276, Russia K. A. Timiryazev Institute of Plant Physiology RAS, IPP RAS Moscow Russia; 4 Key Laboratory of Algal Biology, Institute of Hydrobiology, Chinese Academy of Sciences, Wuhan 430072, China Institute of Hydrobiology, Chinese Academy of Sciences Wuhan China

**Keywords:** Freshwater diatoms, hot spring, monoraphid, taxonomy

## Abstract

During the investigation of the freshwater diatoms from Tibet, a monoraphid species was observed from a hot spring near Anduo County, located on a plateau in the central portion of Tibet. This species shares the diagnostic features of *Crenotia*, such as the valve bent along the transapical axis, striae uniseriate to biseriate from centre to the apices and areolae with special structures located at the end of each stria. We compared the morphological characters of this new species with the others in this genus and show it to be new; it is named *Crenotiatibetia***sp. nov.** This species has small valves with slightly protracted ends with nearly capitate apices, lanceolate axial area, central area unilaterally expanded to the margin, striae uniseriate to biseriate, but, in some valves, the striae are only uniseriate. Areolae are round small to irregular in shape and, at the end of each stria, there is a horseshoe-shaped areola present. Observations of developing valves show all the striae begin biseriate, then they become covered by silica to form uniseriate striae. Comparisons are made amongst the species in this genus and with genera assigned to the Achnanthidiaceae.

## ﻿Introduction

Raphid diatoms that possess a raphe on only one of the valves are very diverse and recent systematic revisions of this group have led to a marked increase in the number of genera, from two (e.g. Hustedt (1930); Patrick and Reimer (1966)) to 27 genera (De Stefano and Marino 2003; Wojtal 2013; Kulikovskiy et al. 2020a; Al-Handal et al. 2021; Ge et al. 2022). Traditionally, these genera have been assigned to one of three families, AchnanthaceaeKützing (1844), CocconeidaceaeKützing (1844) or Achnanthidiaceae Mann in Round et al. (1990). Recent phylogenetic studies have shown these groups to be unrelated, widely dispersed across the raphid diatom tree of life (Thomas et al. 2016; Kulikovskiy et al. 2019). Even genera within the Achnanthidiaceae have been shown to be non-monophyletic (Kociolek et al. 2019; Kulikovskiy et al. 2019).

*Crenotia* was established in 2013 by Wojtal, when species assigned to it were split from the genus *Achnanthidium*. The type species of *Crenotia*, *C.thermalis* (Rabenhorst) Wojtal, was originally described as a species of *Achnanthidium* (as *A.thermalis* Rabenhorst) and then assigned to the genus *Achnanthes* (as *Achnanthesthermalis* (Rabenhorst) Schoenfeld). Eight species have been suggested to belong to *Crenotia* so far. Like other monoraphid diatoms, *Crenotia* has heterovalvar frustules; however, it is distinguished from other monoraphid genera by lacking a cavum on both valves, having biseriate or uniseriate striae, presence of specialised structures at the end of each stria and no ornamentation on the girdle bands. This genus has been reported to have a worldwide distribution (Rioual et al. 2019), usually being found from benthic or periphytic habitats in lakes, springs and swamps and preferring neutral to alkaline waters (Stockner 1968; Hindáková 2009; Wojtal 2013; Kulikovskiy et al. 2016; Coste et al. 2019; Rioual et al. 2019; Wetzel et al. 2019; Liu et al. 2020).

Tibet is one of the biodiversity hotspots of the world and, in this region, the biodiversity of *Crenotia* is also relatively high, with five of the eight species of the genus being reported from Tibet. These species include *C.gibberula* (Grunow) Wojtal, *C.grimmei* (Krasske) Wojtal, *C.hedinii* (Hustedt) Rioual, Ector & Wetzel, as well as three species that are endemic to Tibet, namely, *C.hedinii* (Hustedt) Rioual, *C.distincta* Liu, Kociolek & Xie and *C.oblonga* Liu, Kociolek & Xie (Rioual et al. 2019; Liu et al. 2020). During the investigation of freshwater biodiversity of the Tibetan Plateau, samples were collected from a hot spring in Anduo County, specifically, Nagqu City. One species was observed with light microscopy (LM) and scanning electron microscopy (SEM), based on its morphological features, demonstrated to be a new species belonging to the genus *Crenotia*. Herein we describe this Tibetan diatom as new to science.

## ﻿Materials and methods

Samples were collected from Tibet, during a biodiversity investigation initiated in 2021. Benthic diatoms were collected from Anduo County, Nagqu City, which is located in about the middle of Tibet. Samples were taken from a hot spring located at 31°40′51.24″N, 91°51′20.52″E and 31°40′52.32″N, 91°51′20.52″E, at an elevation of 4570 m above sea level. At the time of collection, the water temperature was around 20 °C, pH ranged from 6.55 to 7.77, conductivity ranged from 2790 to 3200 μS∙cm^-1^(determined by YSI 6920 multiparameter probe). This locality has a cold climate, with dry, windy and cold weather and an annual precipitation of only ca. 100–200 mm.

Samples were fixed with 4% formaldehyde in the field. The samples were cleaned with nitric acid (HNO_3_), then washed and settled using distilled water until the pH was neutral. For LM observations, cleaned diatoms were mounted to make permanent slides with Naphrax. These permanent slides were examined with a Zeiss Imager A2 microscope, equipped with a digital camera (AxioCam MRc 5) and observed with DIC (differential interference contrast) optics (Zeiss, Jena, Germany at Harbin Normal University).

For SEM observations, cleaned material was air-dried and coated with gold-palladium and observations made with a Hitachi S-4800 field emission SEM (Hitachi, Tokyo, Japan at Harbin Normal University) at an operating voltage of 15 kv. Diatom images were compiled with Photoshop 7.0. The holotype slides are deposited in the Key Laboratory of Algal Biology, Institute of Hydrobiology, Chinese Academy of Sciences, Wuhan, China and isotype slides are kept in the College of Life Science and Technology, Harbin Normal University, Harbin, China. Terminology used in the description were referenced by Round et al. (1990), Kingston (2003) and Kulikovskiy et al. (2022).

## ﻿Results

### ﻿ Crenotia tibetia

Taxon classificationPlantaeCocconeidalesAchnanthidiaceae

Liu & Kociolek sp. nov.

F9646BFE-311F-5731-A588-519ACA4C6194

**Figure 1.**
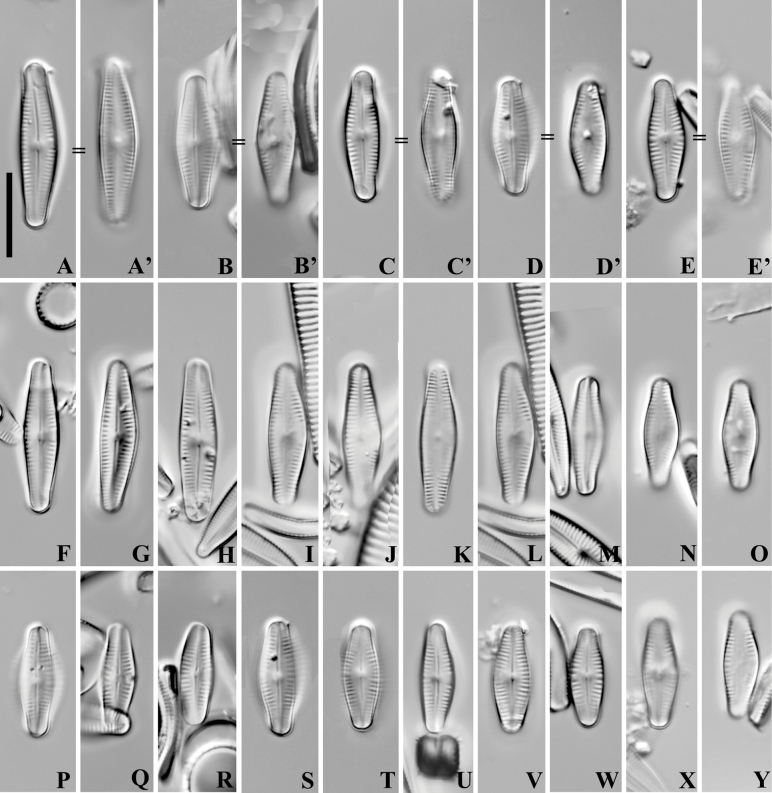
*Crenotiatibetia* sp. nov., LM. Raphe and rapheless valves from the type population. A, A’ illustrations of the holotype. “=” means the raphe valve and rapheless valve are from the same frustule. Scale bar: 10 μm.

**Figure 2.**
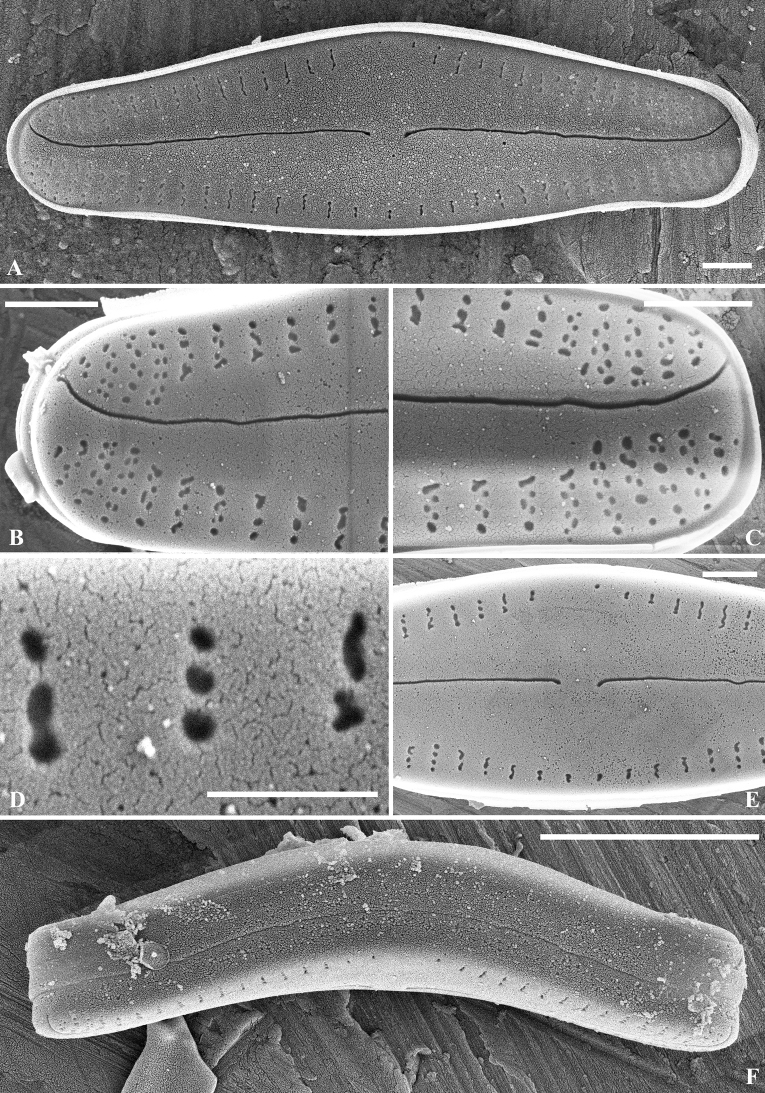
*Crenotiatibetia* sp. nov. SEM, external view of the raphe valve **A** external view of the whole valve **B, C** apices of the valve **D** magnification of areolae **E** central area of the valve **F** girdle view of the valve. Scale bar: 1 μm (**A, B, C, E, F**); 500 nm (**D**).

**Figure 3.**
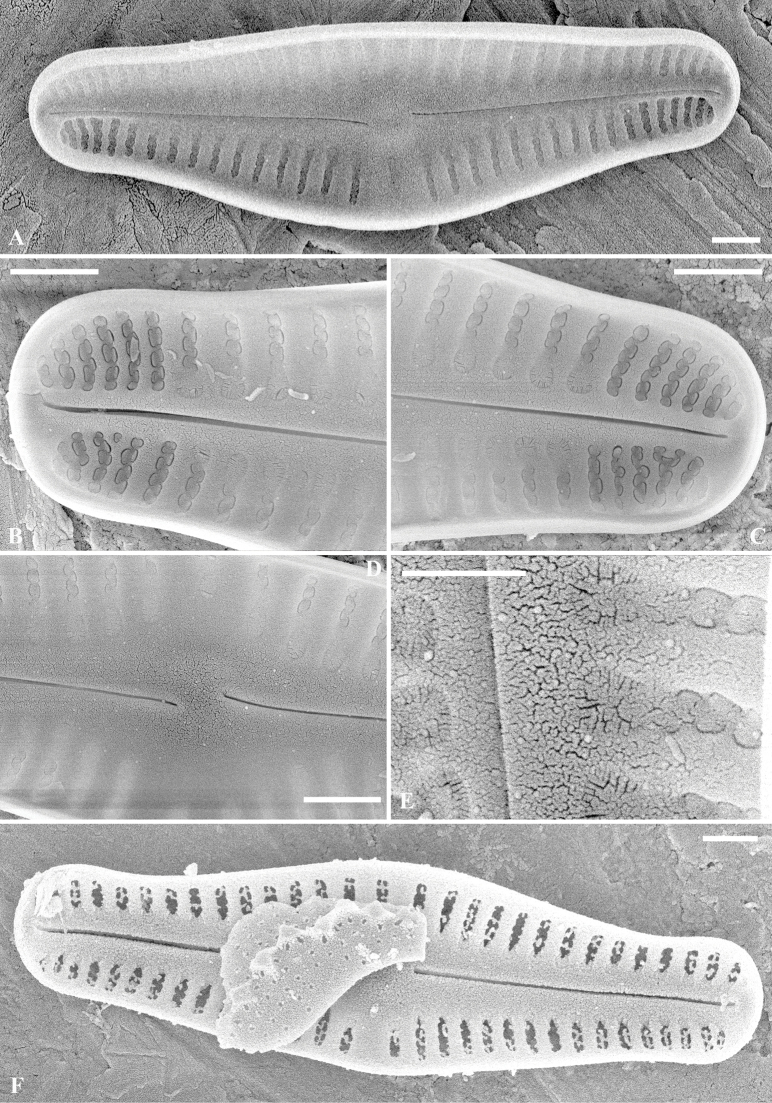
*Crenotiatibetia* sp. nov. SEM internal view of the raphe valve **A** internal view of the whole view **B, C** apices of the valve **D** central area of the valve internally **E** magnification of the areolae, showing the horseshoes areola at the end of striae **F** internal view of a developing valve. Scale bar: 1 μm (**A, B, C, D, F**); 500 nm (**E**).

**Figure 4.**
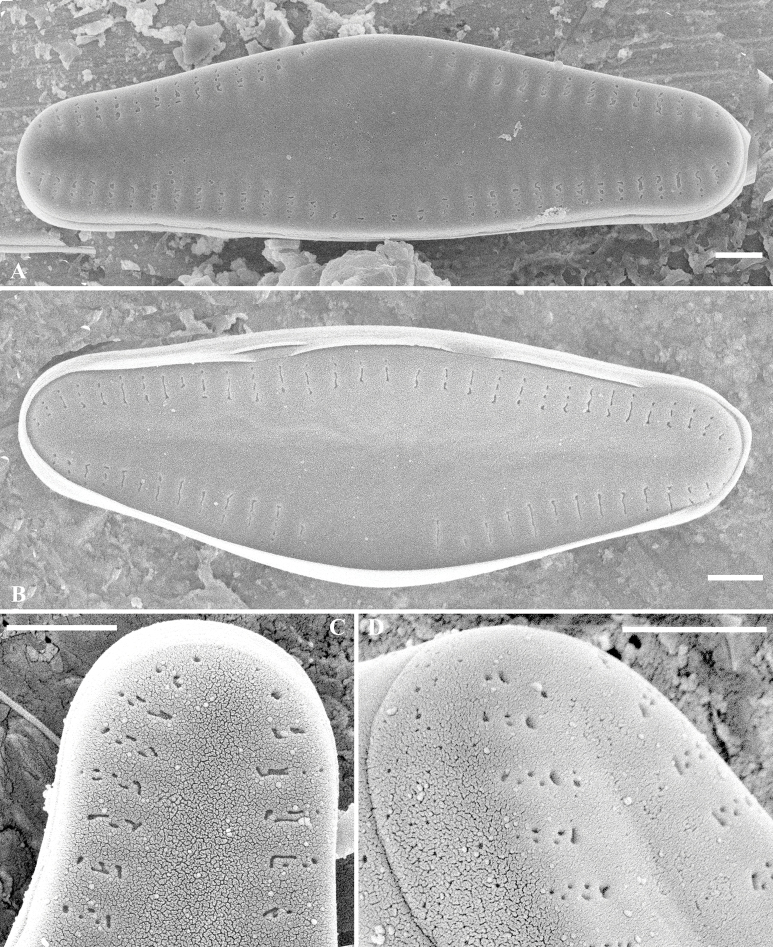
*Crenotiatibetia* sp. nov. SEM external view of the rapheless valve **A, B** external view of whole valve **C, D** apices of the valve, showing the areolae. Scale bar: 1 μm.

**Figure 5.**
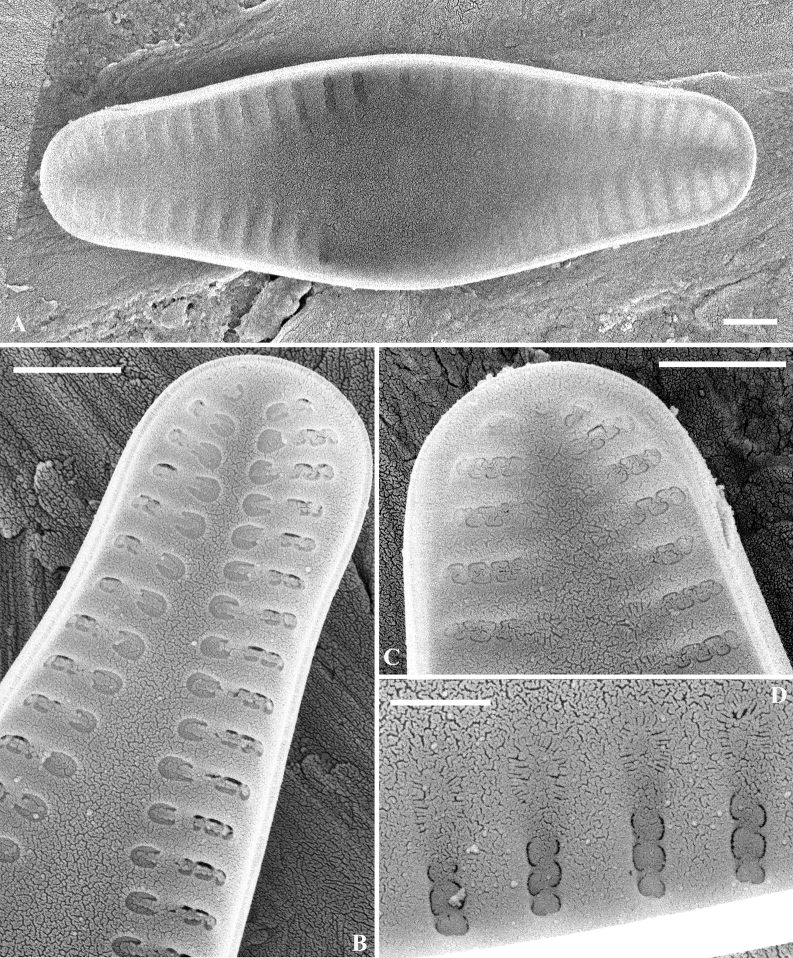
*Crenotiatibetia* sp. nov. SEM internal view of the rapheless valve **A** internal view of the whole valve **B** apex of the developing valve **C** apex of the valve **D** magnification of the areolae, showing the horseshoe-shaped structure at the end of the areolae. Scale bar: 1 μm.

Figs 1 , 2 , 3 , 4 , 5

#### Holotype.

Slide THXZ2021BYQX1–4#, the holotype specimen circled on the slide, illustrated here as Fig. 1A and 1A’; isotype, slide QX1–4#, illustrated here as Fig. 1C and 1C’.

#### Type locality.

China. Tibet, Anduo County, Nagqu City, hot spring, periphyton, 31°41'51.24"N, 91°51'20.52"E, 4570 m a.s.l., collected by Huan Zhu, 31 January 2021.

#### Description.

LM (Fig. 1). Frustule slightly bent along the transapical axis (“V” shaped), monoraphid, with raphe valve concave, rapheless valve convex. Valve lanceolate with slightly protracted ends, slightly asymmetrical to the apical axis, apices nearly capitate. Length 11.8–19.7 μm, breadth 4.1–5.3 μm (n = 30). Raphe valve: straight raphe positioned in the middle of the valve, axial area lanceolate, with asymmetrical, rectangular to rhombic central area. Striae slightly radiating in the centre, becoming parallel towards the apices. Rapheless valve: Axial area lanceolate, central area expanded unilaterally to the margin. Striae 19–21 per 10 μm on both valves.

SEM (Figs 2–5). Raphe valve: Externally, raphe straight, proximal raphe ends slightly deflected to the same side, with distal raphe ends curved to the other side. Axial area lanceolate, nearly 1/3 of the valve width, formed by short striae along the margin. The 3 – 4 striae near the apices are biseriate and become uniseriate towards the valve centre. Areolae openings round to elongate, to irregularly-shaped externally. Internally, proximal raphe ends slightly bent to opposite side, helictoglossae slightly elongated. Areolae covered by hymens, forming two rows of “C”-shaped openings for each stria. Along the axial area, at the end of each stria, there is one horseshoe-shaped structure, open with fine radiating slit-like openings. One developing valve was observed; all the striae were biseriate.

Rapheless valve: Internally, axial area lanceolate, narrow at the apices and becoming wider towards the centre, centre area enlarged unilaterally and reaching the margin. Striae uniseriate to biseriate, mostly biseriate at the apices and becoming uniseriate at the centre. Areolae openings round to irregular in shape. Mantle and girdle bands without ornamentation.

Internally, the axial area is lanceolate, wide in the centre and enlarged at one side extending to the margin. Areolae were occluded by hymens with double rows of “C”-shaped openings; at the end of each stria, there is horseshoe-shaped structure, with fine slit-like openings. A developing valve was observed, all the striae were biseriate, with a “C- shaped structure at the end of each stria.

#### Etymology.

Named after the type locality from which it was found.

## ﻿Discussion

Based on the morphological features of the valve and striae structure, this new species appears to be best placed in the genus *Crenotia*. This small genus currently is known to have nine species, including eight previously-described taxa. The previously-described taxa are: *C.angustior* (Grunow) Wojtal, *C.distincta*, *C.hedinii*, *C.oblonga*, *C.rumrichorum* (Lange-Bertalot) Wojtal, *C.thermalis*, *C.gibberula* and *C.grimmei*.

In comparing this new species with other known taxa (Table 1), *C.angustior* differs by its small frustule and capitate ends. *Crenotiahedinii* was formally transferred to *Crenotia* by Rioual et al. (2019); it has more slender valves and more acutely-rounded ends than our new species.

**Table 1.** Comparison of morphological characteristics of *Crenotiatibetia* sp. nov. and closely related taxa.

	*C.tibetia* sp. nov.	*C.angustior*	*C.distincta*	*C.gibberula*	*C.grimmei*	*C.hedinii*	*C.oblonga*	*C.rumrichorum*	*C.thermalis*
Length (μm)	11.8–19.7	10–19.5	14.0–31.5	13.5–39.5	16–24	7–30	8.0–21.0	12–13.4	7–34
Breadth (μm)	4.1–5.3	4.1–5.2	5.0–8.0	3.6–8	3.6–5	3.2–4.6	4.0–6.0	3.4–4	3.3–5.5
Valve shape	lanceolate	linear to lanceolate	lanceolate	rhombic-shaped	linear	lanceolate	elliptical-lanceolate	narrow lanceolate	elliptical-lanceolate or linear
Valve apices	capitate	small capitate	rounded	capitate	capitate	acutely rounded	rounded	acutely rounded	rounded
Striae	Slightly radiate to parallel / uni to biseriate	Slightly radiate / biseriate	Radiate / multiseriate	slightly radiate	slightly radiate	Slightly radiate / uni to biseriate	Parallel / bi to triseriate	Radiate / uni to biseriate	almost parallel / uni to biseriate
**Raphe valve**									
Axial area	lanceolate	narrow lanceolate	very narrow at the apices, broadly lanceolate in shape	lanceolate	narrow lanceolate	narrow lanceolate	very narrow at the apices, broadly lanceolate in shape	lanceolate, both ends are slightly curved ipsilaterally	linear
Central area	asymmetrical, rectangular to rhombic	small rectangle	absent	obviously enlarged	rectangular	small	absent	small rectangle	rectangular
Raphe	straight	filiform, straight	filiform, straight	filiform, straight	filiform, straight	filiform, straight	filiform, straight	filiform, slightly curved	straight, slightly curved at the end
Striae / 10 μm	19–21	12–16	17–18	14–32	20–22	17–25	22–26	25–27	20–26
**Rapheless valve**									
Axial area	lanceolate	narrow lanceolate	broadly lanceolate	narrow lanceolate	narrow lanceolate	broadly lanceolate	broadly lanceolate	broadly lanceolate	needle lanceolate
Central area	expanded unilaterally to the margin	absent	absent	absent	absent	asymmetry	absent	absent	absent
Striae / 10 μm	19–21	14–18	17–19	12–30	18–22	17–25	22–26	25–27	18–20

*Crenotiagrimmei*, *C.gibberula* and *C.rumrichorum*, the former two species being designated as synonyms of *Achnanthesthermalis* (Rabenhorst) Schoenfeld (Krammer and Lange-Bertalot 2004), are the type species of *Crenotia*. All these three species were transferred to *Crenotia* by Wojtal (2013) when the genus was established.

*Crenotiagrimmei*, first reported by Krasske (1925), was originally named *Achnanthesgrimmei* Krasske; however, Lange-Bertalot and his colleagues rechecked the type and lectotype (Lange-Bertalot and Ruppel (1980), tafel 2, figs 46–50; Lange-Bertalot et al. (1996), tafel 4, figs 8–13) and suggested it is synonymous with *A.thermalis*. *Crenotiagibberula* was described originally as *Achnanthesgibberula* Grunow in Cleve & Grunow. Lange-Bertalot and Ruppel (1980) suggested that *A.grimmei* and its varieties should be considered synonymous with *A.gibberula* and illustrated *A.gibberula* as a morphologically variable taxon (Håkansson 1982). Although Lange-Bertalot and Ruppel (1980) presented the type material of *Crenotiagrimmei* and *C.gibberula*, it is hard to suggest that they belong to the same species, at least based on LM morphology. Based on the illustrations of Lange-Bertalot and Ruppel (1980), Lange-Bertalot et al. (1996) and Wojtal (2013), we can separate *C.thermalis*, *C.grimmei* and *C.gibberula*, based on the morphology of the valves. *Crenotiathermalis* has elliptical-lanceolate or linear valves and the raphe valve face is flat, slightly convex in the central area and the rapheless valves are concave along the apical axis; *C.grimmei* has linear valves with protracted ends that form capitate apices; *C.gibberula* has more rhombic-shaped valves and they have capitate apices and the centre of the valves is obviously enlarged.

LM and SEM images were also presented by Håkansson (1982, plate I: 3–6) for *A.gibberula*, but the striae showed a “macroareolae”-like structure on both valves, similar to those shown in the genus *Madinithidium* Witkowski, Desrosiers & Riaux-Gobin (Desrosiers et al. 2014) or, probably, similar to developing valves of *Achnanthidium* species. Okuno (1974, pl. 855–856), also showed a SEM of *A.grimmei*, but, based on the stria pattern and areola structure, the specimen presented was more similar to *Achnanthidium* rather than *Crenotia*.

Compared with the former three species, *C.tibetia* is morphologically most similar to *C.grimmei*, but *C.tibetia* has a more highly deflected frustule about the transapical axis, the raphe valve is more concave and is larger than *C.grimmei* (Lange-Bertalot et al. (1996) report length 13.0–16.7 μm, breadth 3.6–4.7 μm, striae 16–19/10 μm, for this taxon), striae are denser on both valves, with shorter capitate ends and axial area are wider on the rapheless valves.

*Crenotiagrimmei* and *C.gibberula* were also recorded in Tibet by Zhu and Chen (2000), as *Achnanthesgrimmei* and *A.gibberula*, respectively, plate 46: 11–14), but, based on the published line drawings, their specimens do not match well either of these species.

The morphology of *C.rumrichorum* was observed in detail by Hindáková (2009) as Achnanthesthermalisvar.rumrichorum. Based on the structure, this species was found to belong to the genus *Crenotia*. *Crenotiarumrichorum* has more acute apices, distinguishing it from *C.tibetia*.

Within the genus, *C.distincta* and *C.oblonga* are endemic to Tibet so far and these two species have chambered and multiseriate striae on both valves, which easily distinguish them from our new species. However, these two species do not share the typical features of *Crenotia* and the valve structure resembles the genus *Haloroundia* Diaz & Maidana (2006), a monotypic genus described from Chile. The differences between *Crenotia* and *Haloroundia* can be seen in terms of striae structure, raphe system and degree of flexure of the frustules, but further investigations on the relationships between these two genera are warranted.

The work from Lake Baikal (Kulikovskiy et al. 2013, 2020a, b) established many new genera within Achnanthidiaceae. In research on monoraphid diatoms, curvature of the valve, valve shape and raphe system have been considered as critical features used to separate genera within this family (e.g. Yu et al. (2019); You et al. (2021)). However, molecular data showed the raphe number of frustules does not play such an important role in diatom taxonomy and its reduction or loss occurred many times during the evolution of raphid diatoms (Kulikovskiy et al. 2016). Features such as the “cavum” seem to have played important roles in the evolution of this group. Both morphological features and molecular data were used to identify this group and recognise: 1) species with a sinus; 2) species with a cavum; 3) species without these features (Kulikovskiy et al. 2022). Since more and more “intermediate species” between genera have been observed (You et al. 2021), the relationships between some uniseriate genera, such as *Achnanthidium*, *Gomphothidium* and *Psammothidium* (Round et al. 1990; Bukhtiyarova and Round 1996; Kociolek et al. 2021); and multiseriate genera such as *Platebaikalia*, *Lemnicola* and *Haloroundia* (Diaz and Maidana 2006; Kulikovskiy et al. 2020b) and those with macroareolae, such as *Scalariella*, *Madinithidium*, *Karayevia* and *Kolbesia* (Riaux-Gobin et al. 2012; Desrosiers et al. 2014; Kulikovskiy et al. 2022), appear to be in need of revision. Investigations with formal analyses of both morphological and molecular data may clarify the systematic position and diagnostic features amongst these genera.

## Supplementary Material

XML Treatment for Crenotia tibetia

## ﻿Additional information

### Author ORCIDs

Xinyuan Na https://orcid.org/0009-0005-1425-7610

John Patrick Kociolek https://orcid.org/0000-0001-9824-7164

Maxim Kulikovskiy https://orcid.org/0000-0003-0999-9669

Fengyang Sui https://orcid.org/0000-0002-6734-6570

Guoxiang Liu https://orcid.org/0000-0001-8565-2363

### Data availability

All of the data that support the findings of this study are available in the main text.
